# Melanosis coli: a rarity in digestive endoscopy

**DOI:** 10.11604/pamj.2013.16.86.3331

**Published:** 2013-11-09

**Authors:** Ihsane Mellouki, Houda Meyiz

**Affiliations:** 1 University of Sidi Mohammed Ben Abdellah, Faculty of Medicine and Pharmacy, Department of gastroenterology C, Fez, Morocco

**Keywords:** Melanosis coli, colonic mucosa, endoscopy

## Image in medicine

A 61-year-old patient underwent endoscopic exploration for anemic syndrome. His medical history revealed that he had been taking a drug based on a mixture of medicinal herbs as laxative for nine years. This product consists of anthranoid-containing laxatives as aloe, senna, rhubarb, cascara and frangula. Endoscopy revealed diffuse dark brown pigmentation throughout his colon, which is compatible with the melanosis coli. Further, an unpigmented polyp measuring 8 mm in diameter was seen in the right colon. Macrophages laden with brownish pigment in the lamina propria, were found in all biopsies of the colon. Histopathological examination of the polyp showed a tubulovillous adenoma with low-grade dysplasia. Melanosis coli is a disease characterised by a brownish pigmentation of the colonic mucosa. It is well known that anthranoid containing laxatives, widely used for constipation, are frequently the cause. Anthraquinones have a direct toxic effect on the epithelial cells of the colon that results in the production of lipofuscin, the dark pigment seen in macrophages in melanosis coli. Long-term use of anthranoids is generally believed to be necessary to cause melanosis coli. However, it was established that this condition can develop within periods varying from only 3 to 13 months. The question if melanosis coli predisposes for colorectal neoplasia is discussed controversially. An association of melanosis coli between colorectal adenomas, but not colorectal carcinomas, is under discussion. Melanosis colis is reversible, disappearance of the pigment generally occurs within a year after a patient stops taking anthraquinone.

**Figure 1:**
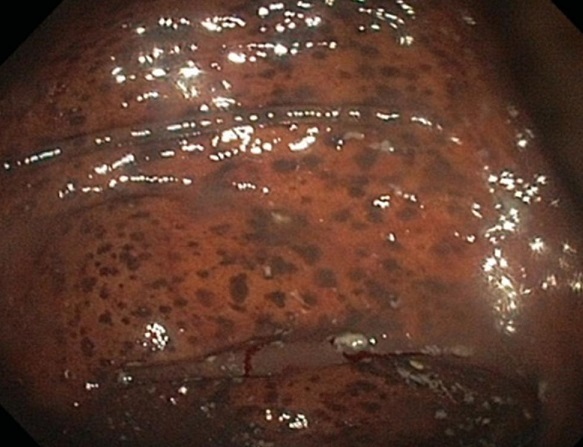
Black Pigmentation of colonic mucosa

